# Hematopoietic Stem Cell Mobilization and Homing after Transplantation: The Role of MMP-2, MMP-9, and MT1-MMP

**DOI:** 10.1155/2012/685267

**Published:** 2012-03-04

**Authors:** Neeta Shirvaikar, Leah A. Marquez-Curtis, Anna Janowska-Wieczorek

**Affiliations:** ^1^Research & Development, Canadian Blood Services, 8249 114th Street NW, Edmonton, AB, Canada T6G 2R8; ^2^Department of Medicine, University of Alberta, Canadian Blood Services Building, 8249 114th Street NW, Edmonton, AB, Canada T6G 2R8

## Abstract

Hematopoietic stem/progenitor cells (HSPCs) are used in clinical transplantation to restore hematopoietic function. Here we review the role of the soluble matrix metalloproteinases MMP-2 and MMP-9, and membrane type (MT)1-MMP in modulating processes critical to successful transplantation of HSPC, such as mobilization and homing. Growth factors and cytokines which are employed as mobilizing agents upregulate MMP-2 and MMP-9. Recently we demonstrated that MT1-MMP enhances HSPC migration across reconstituted basement membrane, activates proMMP-2, and contributes to a highly proteolytic bone marrow microenvironment that facilitates egress of HSPC. On the other hand, we reported that molecules secreted during HSPC mobilization and collection, such as hyaluronic acid and thrombin, increase MT1-MMP expression in cord blood HSPC and enhance (prime) their homing-related responses. We suggest that modulation of MMP-2, MMP-9, and MT1-MMP expression has potential for development of new therapies for more efficient mobilization, homing, and engraftment of HSPC, which could lead to improved transplantation outcomes.

## 1. Hematopoietic Stem/Progenitor Cells (HSPCs) Transplantation

HSPC transplantation is a clinical procedure in which HSPCs capable of reconstituting normal bone marrow (BM) function are administered intravenously to a patient who has undergone preparative regimens including chemotherapy and/or irradiation. Approximately 60,000 autologous and allogeneic HSPC transplants are performed annually worldwide to treat various cancers and diseases of the blood and immune system [1]. During steady-state homeostasis, approximately 0.06% of BM HSPCs circulate continuously in the peripheral blood (PB) [2], but this number can be increased with the use of chemotherapeutic drugs (e.g., cyclophosphamide) and/or growth factors and cytokines (e.g., granulocyte colony-stimulating factor (G-CSF)) that “mobilize” HSPC from BM into the PB [3]. Currently mobilized (m)PB HSPC collection has almost replaced BM harvest for autologous and most allogeneic transplantations because it is relatively easy to collect by apheresis in an outpatient setting and because engraftment after transplantation is faster. G-CSF is the most commonly used mobilizing agent in the clinic with regimens using 10 *μ*g/kg/day for five days when used alone, or 10 to 14 days when used in combination with chemotherapeutic agents [3]. Randomized trials of mPB transplantation have shown that neutrophil and platelet engraftment generally occurs at a median of 9–14 days compared to 21 days with BM [4]. This has been attributed to the higher number of HSPC collected and transplanted. A limitation of mPB HSPC transplantation is that patients' responses to G-CSF vary: 5–10% of allogeneic normal donors mobilize poorly and up to 40% of autologous patients fail to mobilize depending on their disease and the intensity/number of prior chemotherapy regimens [5]. Hence, elucidation of the molecular mechanisms of HSPC mobilization could lead to more efficient mobilizing agents and development of better protocols.

An alternative source of HSPC is cord blood (CB) obtained at the time of childbirth, after the umbilical cord has been detached from the newborn. Since the first CB transplant in 1988, more than 30,000 CB transplants have been performed worldwide in pediatric and adult patients [1, 6]. CB has several advantages over BM and mPB as source of HSPC for transplantation. CB contains lower numbers of more immature, immunocompetent T cells and thus requires less stringent HLA matching; this means that a mismatch at one or two loci can be tolerated without significant increase in graft versus host disease (GvHD) or decrease in overall survival [6, 7]. However, the main limitation of CB transplantation is the low CD34^+^ cell dose available in one CB unit which is generally insufficient to support engraftment in adult patients. Retrospective analyses of outcomes of CB and BM transplantation in adults have reported delayed neutrophil engraftment: 27 days with CB versus 18 days with BM and platelet engraftment: 60 days with CB versus 29 days with BM [7]. Currently efforts are being made to increase CB cell dose in order to speed up engraftment and hematopoietic recovery. Strategies to use more than one CB unit [7] or to expand CB CD34^+^ cells ex vivo [8], however have met with limited success. A more comprehensive knowledge of CB HSPC biology and the mechanisms of their homing is expected to improve CB transplantation outcomes.

## 2. Mobilization of HSPC

During homeostasis, continuous traffic of HSPC between the BM and PB contributes to normal hematopoiesis. The ability to enhance these physiological processes and, for example, enforce egress (mobilization) of HSPC from the BM to circulation has been invaluable in clinical transplantation [9]. Several cytokines (G-CSF, granulocyte macrophage-CSF, Flt-3 ligand, interleukin (IL)-8, and stem cell factor (SCF)/kit-ligand) and chemokines (stromal cell-derived factor (SDF)-1 and GRO*β*) can trigger mobilization in varying degrees [3, 10]. We recently demonstrated that G-CSF also increases plasma hepatocyte growth factor (HGF) levels in mobilized patients and expression of its receptor c-Met in HSPC and myeloid cells, suggesting that GCSF-mediated HSPC mobilization occurs in part through the HGF/c-Met axis [11].

Another important axis that influences mobilization is the SDF-1/CXCR4 axis. This axis is essential for retention of HSPC in the BM, and perturbation of the SDF-1 gradient in the BM as well as a decrease in the responsiveness of HSPC to SDF-1 leads to mobilization [12].

The proteases carboxypeptidase M (CPM) [13] and CD26 [14] cleave the C-terminus of SDF-1 resulting in attenuated chemotactic responses of HSPC; moreover, G-CSF-induced mobilization is impaired in CD26-deficient mice [14]. Desensitization of CXCR4 by the urokinase-mediated plasminogen activation system during G-CSF-induced mobilization has been demonstrated [15]. In murine studies, SDF-1 concentration in the BM decreased following G-CSF administration and correlated with mobilization, suggesting that a physiological drop in SDF-1 level in the BM is a critical step in HSPC mobilization [12, 16]. A decrease in BM SDF-1 levels has been reported to coincide with a peak of proteolytic activity of neutrophil elastase (NE), cathepsin G (CG), and matrix metalloproteinase (MMP)-9 [17]. Both SDF-1 and CXCR4 are targets of degradation by NE, CG, MMP-9, MMP-2, and membrane type (MT)1-MMP [18–20] and can be inactivated by proteolytic cleavage. The roles of these MMPs will be discussed in detail in subsequent sections.

Recent studies have demonstrated that thrombolytic agents such as microplasmin, tenecteplase, and recombinant tissue plasminogen activator enhance G-CSF-induced mobilization in murine models [21]. Adhesion molecules also play an important role in the retention of HSPC in the BM. Very late antigen (VLA)-4 is expressed by HSPC and its ligand, vascular cell adhesion molecule (VCAM)-1, is constitutively expressed by endothelial and stromal cells [22]. Disruption of the VCAM-1/VLA-4 axis with a small molecule inhibitor of VLA-4 resulted in the mobilization of more HSC over basal levels [23]. CD44, a polymorphic integral membrane glycoprotein, binds to several extracellular matrix (ECM) components such as hyaluronic acid (HA), fibronectin, and collagen and mice treated with anti-CD44 antibody or lacking CD44 exhibit impaired mobilization in response to G-CSF [24].

Components of innate immunity also participate in G-CSF-induced HSPC mobilization [25]. During G-CSF-induced mobilization, the complement cascade (CC) is activated by the classical pathway. However, in the early steps of the CC, C1q, C3, and its cleavage fragments C3a and _desArg_C3a increase retention of HSPC in BM while in the later steps of the CC, C5, and its cleavage fragments C5a and _desArg_C5a enhance HSPC mobilization and their egress into PB [26–28].

## 3. Homing of HSPC

The success of clinical HSPC transplantation relies on the inherent ability of transplanted HSPC to home efficiently to the BM niche and engraft. Interactions between HSPC and their niches that are disrupted during mobilization need to be reestablished during their homing to the BM and its repopulation. Previously it was believed that mobilization and homing were mirror images of each other; however, emerging evidence now suggests that this is not the case, although both processes involve many of the same adhesive and chemotactic interactions. The homing of HSPC to BM is a rapid process occurring within hours after their transplantation and is a prerequisite for their repopulation and engraftment [29]. Homing involves three consecutive steps: extravasation from PB through the BM endothelium, migration through stroma, and lodgement into niches.

The extravasation of circulating HSPC within the BM requires a set of molecular interactions that mediates the recognition of circulating HSPC by the endothelium of BM sinusoids [30]. The mechanisms of HSPC extravasation are similar to those of leukocytes into inflamed tissues in that they are mediated by adhesion molecules. The BM endothelium constitutively expressing P-selectin, E-selectin, and VCAM-1 mediates rolling and tethering of HSPC to the blood vessel wall prior to their extravasation [31]. HSPC expressing CD44 and VLA-4 receptors interact with their cognate ligands on the endothelial surface. The coordinated action of these adhesion molecules is triggered by SDF-1 presented at the surface of endothelial cells. SDF-1 mediates activation of lymphocyte function-associated antigen-1, VLA-4 and VLA-5, converting the rolling of HSPC into stable arrest on the endothelium [32].

SDF-1—CXCR4 signalling is critical in the regulation of HSPC homing, engraftment, and retention in the BM. Blockade of CXCR4 was shown to inhibit HSPC homing and engraftment, whereas overexpression of CXCR4 by CB and mPB CD34^+^ cells leads to increased SDF-1 induced in vitro migration and homing in NOD/SCID mouse models [33, 34]. We recently showed that mPB CD34^+^ cells that had higher responsiveness to SDF-1 had high CXCR4 expression and could compensate for a lower CD34^+^ cell dose in achieving faster hematopoietic engraftment after transplantation [35]. SDF-1 binding to CXCR4 activates phosphatidylinositol 3-kinase (PI3K), the phospholipase C-*γ* (PLC-*γ*)/protein kinase C (PKC) cascade, and p44/42 mitogen-activated protein kinase (MAPK) [36]. SDF-1—CXCR4 signalling also activates the atypical PKC subtype PKC*ζ* which mediates cell polarization, adhesion, MMP-9 secretion, and chemotaxis of CD34^+^ cells [37]. Interactions mediated by the Rho family GTPases (Rac, Rho, and Cdc42) have also been implicated in HSPC homing and engraftment. Following engraftment, deletion of Rac-1 and Rac-2 GTPases led to massive mobilization of HSPC from BM. Knocking out Rac-1 significantly reduced migration towards SDF-1 and attenuated the homing of murine HSC to the endosteum, which is essential for long-term HSC repopulation [38]. We previously demonstrated that Rac-1 colocalization with CXCR4 in membrane lipid rafts of HSPC promotes their in vivo homing in a murine model [39]. Moreover, in vitro treatment of HSPC with supernatants of leukapheresis products (SLPs) or their components, such as C3a or platelet-derived microvesicles (PMVs), modulates the SDF-1—CXCR4 axis and speed up in vivo homing in murine models [39, 40].

Proteases regulate HSPC migration and tissue localization and have been shown to also play important functions in HSPC homing. The roles of proteolytic enzymes particularly MMP-2, MMP-9, and MT1-MMP in this process will be discussed in detail in subsequent sections.

## 4. Matrix Metalloproteinases

MMPs belong to a family of Zn^2+^-binding, Ca^2+^-dependent endopeptidases whose essential function is proteolysis of the ECM, a process that is required in several cellular processes [41]. Currently, 24 human MMPs have been identified that have structural similarities but vary in their expression profiles and substrate specificities. MMPs are classified based on substrate recognition into stromelysins (MMP-3, MMP-10, and MMP-11), matrilysins (MMP-7, MMP-26), gelatinases (MMP-2 and MMP-9), and collagenases (MMP-1, MMP-8, MMP-13, and MMP-14) [42]. Apart from ECM molecules, MMPs act on a whole array of substrates including other proteinases and MMPs, proteinase inhibitors, growth factors, cytokines, chemokines, cell surface receptors, and cell adhesion molecules and regulate many processes such as cell migration, proliferation, apoptosis, angiogenesis, tumor expansion, and metastasis [42–44]. The expression and function of MMPs are regulated at different levels. Generally expressed at low levels, MMPs are upregulated during tissue remodeling, inflammation, wound healing, and cancer progression [45, 46]. They are synthesized as latent enzymes that are either secreted or membrane-anchored. Six MT-MMPs have been identified so far, of which four, MT1-/MMP-14, MT2-/MMP-15, MT3-/MMP-16, and MT5-/MMP-24, have a transmembrane domain while the other two, MT4-/MMP-17 and MT6-/MMP-25, have a glycosylphosphatidylinositol domain [42]. Their membrane anchoring allows them to carry out pericellular proteolysis. MMPs are activated by the proteolytic release of the N-terminal propeptide domain. Once active, they can be inhibited by endogenous tissue inhibitors of metalloproteinases (TIMPs), the reversion-inducing cysteine-rich protein with Kazal motifs (RECK), and tissue factor pathway inhibitor-2 as well as by plasma inhibitor (*α*2-macroglobulin) [47, 48]. Therefore, a balance between MMPs and their inhibitors is important to ECM remodeling in the tissue and in HSPC migration.

The gelatinases MMP-2 and MMP-9 have been extensively studied in cancer and other diseases. MMP-2 is secreted by fibroblasts, endothelial cells, epithelial cells, and transformed cells whereas MMP-9 is produced predominantly by leukocytes [49]. MMP-2 and MMP-9 are required for physiological processes such as ECM remodeling during growth and development, inflammation, wound healing, angiogenesis, and leukocyte mobilization [41, 46]. They are also involved in pathological processes such as cancer, inflammation, and neural and vascular degenerative diseases [45, 46]. Although MMP-2 and MMP-9 are secreted by cells in the developing embryo, mice deficient in these gelatinases are viable. However, mice deficient in MMP-2 exhibit defects in developmental angiogenesis whereas mice deficient in MMP-9 show delayed vascularization and ossification resulting in moderate skeletal abnormalities [49]. MMP-2 and MMP-9 are similar in many respects, but differ in their glycosylation pattern, activation, and substrate specificity. The 92 kDa MMP-9 has two glycosylation sites in the prodomain and the catalytic domain whereas the 72 kDa MMP-2 is a nonglycosylated protein [42]. MMP-2 activation is a cell surface event mediated by the formation of a ternary complex containing MT1-MMP, TIMP-2, and proMMP-2 [42]. The N-terminal domain of TIMP-2 binds to MT1-MMP whereas the C-terminal domain binds the hemopexin domain of proMMP-2. An adjacent MT1-MMP free of TIMP-2 subsequently activates proMMP-2 by cleaving its propeptide domain. On the other hand, MMP-9 activation is mediated by a proteolytic cascade involving MMP-3, MMP-2 and MMP-13 [42]. MMP-3 is activated by plasmin generated from plasminogen by urokinase-type plasminogen activator (uPA) bound to its receptor on the cell surface [42]. Similarly, MMP-2 activates proMMP-13 which then activates proMMP-9 [50]. MMP-2 and MMP-9 share similar proteolytic activities and degrade a number of ECM molecules such as gelatin, collagen types IV, V, and XI, and laminin. In addition MMP-2 also degrades collagen types I, II and III. Both also cleave several non-ECM molecules such as SDF-1, tumor necrosis factor (TNF)-*α*, transforming growth factor (TGF)-*β*, plasminogen, and uPA, among many others [42–44].

MT1-MMP also plays important roles in both physiological and pathological processes. Mice deficient in MT1-MMP exhibit craniofacial dysmorphism, dwarfism, osteopenia, fibrosis of soft tissues, arthritis, and premature death, emphasizing the function of MT1-MMP in ECM remodelling during development [51]. On the other hand, elevated MT1-MMP expression has been observed in a wide variety of cancers including lung, breast, cervical, brain, liver, head, and neck, indicating MT1-MMP's role in tumor progression and metastasis [45]. In addition, it has been implicated in angiogenesis, bone development, atherosclerosis, inflammation, and wound healing [44] by virtue of its ability to degrade several ECM macromolecules including collagens, laminins, fibronectin, aggregan, and fibronectin and to activate proMMP-2 and proMMP-13 [42, 44, 52]. MT1-MMP cleaves CD44, processes *α*_v_ integrin to its mature form, and degrades tissue transglutaminase, thus modifying the immediate cell environment and affecting cellular functions in a variety of ways [44]. MT1-MMP has a domain structure composed of a propeptide region, catalytic domain, hinge region, hemopexin-like domain, and a type I transmembrane domain with a cytoplasmic tail at the C-terminus which anchors it to the cell surface [42]. MT1-MMP is also expressed in its latent form and cleavage of the propeptide region by furin or related protein convertases renders it active. Activation of MT1-MMP takes place in the trans-Golgi network complex during secretion, and the enzyme is expressed in its active form at the cell surface [53]. Active MT1-MMP is inhibited by RECK [54], TIMP-2, TIMP-3 and TIMP-4 but not by TIMP-1 [48]; however, TIMP-2 has a dual role. On the one hand it inhibits MT1-MMP and on the other promotes activation of proMMP-2 [47]. At the cell surface, the 60 kDa active MT1-MMP molecule undergoes self-proteolysis by removal of its catalytic domain which results in an inactive 44 kDa species [55]. A high level of truncated MT1-MMP coincides with high proMMP-2 activation. In migrating tumor cells, MT1-MMP localizes predominantly in the lamellipodia through its interaction with CD44 [56]. It has been reported that this interaction and the consequent shedding of CD44 stimulate cell motility [57]. The hemopexin domain of MT1-MMP interacts with the stem region of CD44 which interacts with F-actin through its cytoplasmic domain. Binding of MT1-MMP with CD44 indirectly links the proteinase to the cytoskeleton and thereby enables its localization to the lamellipodia [58]. MT1-MMP can be internalized by both clathrin-dependent and caveolae-dependent pathways, and recycled back to the surface, and the internalization process is essential for the enzyme to promote cell migration [58]. The redistribution of MT1-MMP to sites of degradation, such as the lamellipodia of endothelial cells and the invadopodia of tumor cells, is highly complex and involves a dynamic interplay between endocytic and exocytic processes [59]. In most cell types surface expression of MT1-MMP is low due to rapid endocytosis resulting in intracellular accumulation of the proteinase in the early and late endosomes. This dynamic regulation suggests that the spatiotemporal recruitment of MT1-MMP to specialized domains plays a critical role in its invasive properties [60]. Various growth factors, chemokines, and inflammatory mediators have been reported to modulate MT1-MMP expression in malignant and normal cells. In the fibrosarcoma cell line HT1080, Rac-1 modulation of MT1-MMP and its processing to its 44 kDa form correlated with proMMP-2 activation [61]. Furthermore, Rac-1 has been demonstrated to promote hemophilic complex formation of MT1-MMP, recruitment to the lamellipodia-rich cell surface, and subsequent proMMP-2 activation [62]. In Lewis lung carcinoma cell line, type 1 insulin growth factor-1 increases invasiveness of these cells through increased MT1-MMP expression via the PI3K/AKT/mTOR pathway [63]. The SDF-1−CXCR4 axis promotes melanoma cell invasion and metastasis by upregulating MT1-MMP through Rac-1 and RhoA-GTPases [64]. Moreover, through activation of Rac-1 and its downstream effector ERK1/2, SDF-1 intracellularly upregulates MT1-MMP; however, when these cells were in contact with Matrigel, a PI3K-dependent transient redistribution of MT1-MMP to the cell surface was observed [65]. In endothelial cells, VEGF-mediated upregulation of MT1-MMP at the mRNA level occurs through the MAPK/JNK pathway, whereas protein expression is regulated by PI3K [66, 67]. MT1-MMP clustering on the cell surface is dependent on cortical actin polymerization which is regulated by PI3K, and this clustering of MT1-MMP has been suggested to be more important than its internalization [59]. In mesenchymal stromal cells (MSCs), we reported that MT1-MMP mediates their chemotactic migration towards SDF-1 and HGF [68]. Increased chemoinvasion of MSC through upregulation of MT1-MMP by cytokines TGF-*β*, TNF-*α*, and IL-1 was also reported [69]. In hematopoietic cells, MT1-MMP has been demonstrated to mediate trans-endothelial migration of monocytes, and the interaction of monocytes with fibronectin and endothelial ligands, such as VCAM-1 and intracellular cell adhesion molecule (ICAM)-1, increased MT1-MMP clustering and localization into membrane protrusions or lamellipodia [70].

## 5. MMPs in HSPC Mobilization

MMPs have been traditionally considered to facilitate cell migration by breaching basement membrane barriers comprised of ECM proteins. The mobilizing agent G-CSF induces neutrophil proliferation, activation, and degranulation with the subsequent release of the serine proteases NE, CG, and MMP-9 into the BM, making it a highly proteolytic microenvironment [17, 71]. MMP-9 has been reported to be elevated in plasma after mobilization with G-CSF or IL-8 [71, 72]. Mobilization is thought to occur predominantly through the action of neutrophils and release of granulocytes from the BM always precedes mobilization of HSPC [73]. However, HSPC themselves contribute to this process by secreting MMP-2 and MMP-9 [74]. We have reported that while BM HSPCs in steady-state do not secrete MMPs, upon stimulation with G-CSF, among other growth factors and cytokines, HSPCs secrete both MMP-2 and MMP-9 leading to their enhanced migration through reconstituted basement membrane (Matrigel) [74]. Both mature leukocytes as well as immature CD34^+^ cells from in vivo G-CSF-mobilized PB highly express MT1-MMP compared to their steady-state (ss)BM and ssPB counterparts. Furthermore, cell surface expression of MT1-MMP in in vivo mobilized polymorphonuclear cells (PMNs) and monocytes was 8 and 12 times higher than in their respective ssPB counterparts. G-CSF exerts its effects on other signalling axes that also result in upregulation of MMP-9 and MT1-MMP. For example, we observed that HGF increases in the plasma of mobilized patients and that G-CSF, through induction of HGF, upregulates MMP-9 and MT1-MMP expression in BM PMN [11]. In addition, activation of the CC by G-CSF triggers a series of reactions generating various bioactive peptides including the C5 cleavage fragments (C5a and _desArg_C5a) which have been shown to play an important role in mobilization [25, 75]. In particular, we recently demonstrated that C5a increases MMP-9 and MT1-MMP expression in PMN and mononuclear cells (MNCs), contributing to a microenvironment that is conducive to the egress of HSPC from the BM [28]. MT1-MMP causes pericellular degradation by processing several ECM components (such as gelatin, fibronectin, laminin, vitronectin, and fibrillar collagens) [41, 43, 52]. This is further substantiated by observation that G-CSF-induced migration of MNC and HSPC through Matrigel (containing collagen type IV, laminin, entactin) is MT1-MMP dependent. MT1-MMP expressed by HSPC activates proMMP-2 secreted by BM stromal cells [76, 77]. Active MMP-2 could subsequently initiate a cascade of activation of other MMPs including MMP-9 and MMP-13, which not only degrade the ECM but also disrupt adhesive interactions between HSPC and their niches. One of the most potent of these interactions is between SDF-1, produced by stromal cells, including endothelial cells, and osteoblasts, and its receptor CXCR4, expressed by HSPC. Recently, we demonstrated that C5a, apart from upregulating MT1-MMP, decreases CXCR4 expression in PMN, which disrupts their chemotaxis towards SDF-1. Moreover, this impaired chemoattraction is partially restored by the potent MT1-MMP inhibitor EGCG (which also inhibits proMMP-2 activation), suggesting that MT1-MMP contributes towards reduced retention of HSPC in the BM microenvironment [28].

In addition to matrix remodeling, MT1-MMP cleaves adhesion molecules, such as CD44 and integrins, and chemokines such as SDF-1. Reduced retention of HSPC in the BM during G-CSF-induced mobilization is also due to a decrease in active SDF-1 in the BM which coincides with peak proteolytic activity [12, 16]. SDF-1 can be cleaved and inactivated by several proteases which are activated during mobilization such as NE, CG, MMP-2 and MMP-9, CD26, CPM, and MT1-MMP [13, 14, 17–20]. MT1-MMP cleavage of SDF-1 results in loss of binding to CXCR4 and reduced chemoattraction for CD34^+^ cells [18]. Therefore, during G-CSF-induced mobilization, upregulation of MT1-MMP expression in HSPC has consequences that could lead to their reduced retention in the BM and resultant egress into PB. 

A low SDF-1 level in the BM during cytokine-induced mobilization has been suggested to result from suppression of SDF-1-producing osteoblasts, and although the mechanism of this phenomenon is not completely understood [78], it has been suggested to occur in light of an observed increase in osteoclast activity [79]. Active bone remodeling takes place during G-CSF mobilization, and increased cathepsin K and MMP-9 secretion by osteoclasts has been implicated in high bone turnover [79, 80]. Similarly, MT1-MMP also plays an important role in bone remodeling as shown by the fact that MT1-MMP^−/−^ mice exhibit severe skeletal defects [51]. MT1-MMP is also known to be expressed by osteoclasts and recently it was demonstrated that MT1-MMP was necessary for macrophage fusion during multinucleated osteoclast formation and differentiation [81]. Accordingly, 8-day-old MT1-MMP^−/−^ mice exhibited impaired osteoclast function, which did not, however, result in increased bone mass since osteoblast function is also compromised in these mice [51, 81]. These results suggest that MT1-MMP could have a dual role in bone development. Nevertheless, the role of MT1-MMP in bone remodeling during G-CSF-induced mobilization requires further investigation. 

It was postulated that MT1-MMP expression in HSPC is regulated by the endogenous inhibitor RECK and that high MT1-MMP and low RECK levels in HSPC resulted in the egress of BM progenitors into circulation [54, 77]. In a recent publication, we provided evidence that MT1-MMP expression on the surface of HSPC is regulated by its incorporation into membrane lipid rafts, and that both MT1-MMP expression and proMMP-2 activation are PI3K-dependent [76]. Moreover, in co-cultures of fibroblasts with BM CD34^+^ cells, proMMP-2 is not activated; however, pre-incubation of CD34^+^ cells with G-CSF highly upregulated MT1-MMP, which was then able to activate proMMP-2 in similar co-cultures. Consistent with this, in co-cultures of stromal cells with mPB CD34^+^ cells that had been transfected with MT1-MMP siRNA, active MMP-2 was not detectable [76]. The MT1-MMP activation of proMMP-2 in the BM microenvironment is important because active MMP-2 not only initiates the activation of other MMPs that play a role in matrix remodeling, but also inactivates SDF-1 and CXCR4 and adhesion molecules, processes which facilitate egress of HSPC from BM niches across the ECM and subendothelial membranes.

MT1-MMP promotes cell motility by pericellular ECM degradation [41, 52, 53]. In this respect we demonstrated the role of MT1-MMP in the migration of CD34^+^ cells and MNC across reconstituted basement membrane as specific inhibition of MT1-MMP by siRNA significantly abrogated their migration [76]. On the other hand, upregulation of MT1-MMP expression in HSPC by G-CSF could lead to their reduced retention in the BM and enhanced egress into PB [77]. The definitive proof of this would involve studies using MT1-MMP^−/−^ mice, but such mice have severe developmental abnormalities resulting in their early death [51]; hence in vivo mobilization experiments in MT1-MMP knockout mice are difficult. However, these experiments have been carried out in chimeric mouse models [77]. 

**Figure 1:**
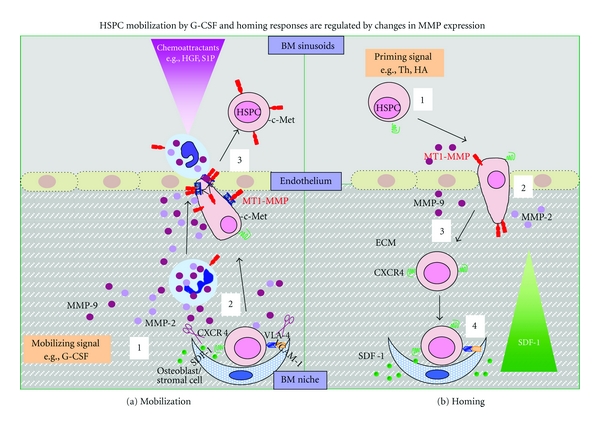
(a) Administration of a mobilizing agent such as G-CSF (1) expands the number of myeloid cells (neutrophils/granulocytes) in the bone marrow and promotes increased expression of proteolytic enzymes (including MMP-2, MMP-9, and MT1-MMP). (2) These enzymes disrupt interactions that retain HSPC in their BM niches (e.g., SDF-1/CXCR4, VCAM-1/VLA-4, kit ligand/c-kitR, ECM). (3) Subsequently, permeabilization of the endothelial barrier occurs with the granulocytes “paving the way” for the egress of HSPC. Chemoattractants in the blood (primarily bioactive lipids such as sphingosine-1 phosphate (S1P) and hepatocyte growth factor (HGF)) further potentiate mobilization of HSPC to the peripheral blood. (b) Priming molecules such as hyaluronic acid and thrombin (1) amplifiy the chemotactic responses of HSPC via CXCR4 towards SDF-1 produced by bone marrow stromal cells and (2) accumulate MT1-MMP on the cell migration front. (3) MT1-MMP promotes activation of MMP-2 that degrades ECM barriers allowing extravasation of HSPC across the sinusoid endothelium. (4) HSPC highly expressing in CXCR4 attaches to the SDF-1-rich endosteal niches of the bone marrow.

The initial cell response to cytokine or chemokine stimulation is the reorganization of the actin cytoskeleton during which several signalling pathways are activated. MT1-MMP has been shown to be modulated by PI3K, MAPK, and Rho family GTPases depending on the cell type and stimulant [64, 82]. We demonstrated that although both PI3K and MAPK are activated in hematopoietic cells upon G-CSF stimulation, MT1-MMP expression and proMMP-2 activation are only PI3K dependent. Furthermore, inhibition of the PI3K-AKT axis also inhibits cell polarization, co-localization of MT1-MMP with F-actin, and trans-Matrigel migration of mPB CD34^+^ cells [76]. Murine HSPC studies have shown that cytokine-mediated lipid raft clustering activated the AKT-FOXO signalling pathway, which is essential for entry into cell cycle, and inhibition of lipid raft formation by M*β*CD led to repression of this pathway and hibernation-like state of HSPC [83]. The recruitment of the regulatory PI3K subunit p85 to lipid rafts after cytokine stimulation is required for activation of the downstream effectors of the PI3K-AKT axis and is dependent on lipid raft integrity [84]. Consistent with these findings, we showed that G-CSF-induced PI3K activation in lipid rafts leads to reorganization of the actin cytoskeleton, recruitment of MT1-MMP into lipid rafts, and proMMP-2 activation [76], which increases the degradation of basement membrane and interstitial matrix in the BM microenvironment and eventually the egress of HSPC into circulation as shown in Figure 1(a). Mobilizing signals such as G-CSF expand the number of myeloid cells in the BM, which secrete proteolytic enzymes (including MMP-2 and MMP-9) that disrupt interactions that retain the HSPC in the BM (e.g., SDF-1/CXCR4, VCAM-1/VLA-4, ECM). Permeabilization of the endothelial barrier occurs with granulocytes “paving the way” for HSPC. Chemoattractants in the blood (e.g., bioactive lipids such as sphingosine 1-phosphate, HGF) further facilitate egress into PB of HSPC [73] (Figure 1(a)).

## 6. MMPs in HSPC Homing

MMPs degrade various ECM molecules and facilitate HSPC transmigration across basement membrane barriers. SDF-1 and other growth factors induce the secretion of MMP-2 and MMP-9, thus facilitating in vitro migration of HSPC across reconstituted basement membrane towards SDF-1 [73]. Incubation of CB-derived HSPC with SCF induces MMP-2 and MMP-9 secretion and homing in NOD/SCID mice [85]. We demonstrated that MT1-MMP mediates migration of CB CD34^+^ cells and megakaryocytic progenitors towards an SDF-1 gradient [86]. Recently another group reported, using a chimeric mouse model, that engraftment levels of MT1-MMP^−/−^ c-Kit^+^ cells were significantly lower than those of wild-type cells, and inhibition of MT1-MMP by monoclonal antibody attenuated homing of human HSPC in a NOD/SCID mouse model [77].

 We have demonstrated that HSPC primed by SLP or their components (fibrinogen, fibronectin, complement C1q, complement C3a, PMV, HA, thrombin) respond better to an SDF-1 gradient [26, 39, 40, 87, 88]. Furthermore, murine HSPC that have been primed with C3a, PMV, or SLP before transplantation engrafted faster in lethally irradiated mice [39, 40, 87]. This priming effect was due to increased incorporation of CXCR4 and Rac-1 GTPase into membrane lipid rafts and to increased MMP-2 and MMP-9 secretion, indicating that the SDF-1—CXCR4 axis cooperates with MMPs during HSPC homing [39].

We recently investigated the effects of two priming molecules, HA and thrombin, on the modulation of MT1-MMP expression and its activity [88]. First, we found that HA and thrombin upregulated MT1-MMP expression in CB HSPC. Secondly, HA- and thrombin-primed chemoinvasion of HSPC towards a low SDF-1 gradient was MT1-MMP dependent. It has been established that the SDF-1—CXCR4 axis promotes the chemotaxis not only of normal but also of tumor cells [70, 89]. For example, the coordinated interaction of CXCR4 and MT1-MMP is required for melanoma cell metastasis to lungs [65]. CXCR4 was required for the initial phases of melanoma cell chemotaxis and their arrival in the lungs, whereas MT1-MMP was necessary for subsequent invasion and dissemination of the tumor. We can therefore speculate that a coordinated interaction between CXCR4 and MT1-MMP is also required by HSPC for their homing to the BM. MT1-MMP expressed in CB HSPC (upregulated by HA and thrombin), activates proMMP-2 secreted by endothelial cells. Active MMP-2 could thereby participate in the extravasation process and facilitate homing by activating other MMPs and degrading ECM barriers [73]. In this respect, MT1-MMP has been shown to facilitate trans-endothelial migration of monocytes through clustering of MT1-MMP at the lamellipodia upon contact with activated endothelial cells or the immobilized endothelial ligands VCAM-1 and ICAM-1 [70]. Priming of homing-related responses of CB HSPC showed that HA and thrombin activated the PI3K-AKT signalling axis and Rac-1 GTPase [88]. MT1-MMP expression and proMMP-2 activation were dependent on both these signalling pathways. We demonstrated that intracellular crosstalk between these pathways leads to signal amplification of a low SDF-1 gradient, leading to enhanced MT1-MMP expression on the cell surface of CB HSPC and increase chemoinvasion towards SDF-1 [88]. Thus, agents such as HA and thrombin that positively regulate the SDF-1—CXCR4 axis may also prime the homing-related responses of HSPC by upregulating MT1-MMP. HSPCs then attach/tether to and extravasate the sinusoid endothelium. MT1-MMP promotes activation of proMMP-2 that degrades ECM barriers. HSPCs are chemoattracted to their BM niches due to amplified chemotactic response towards SDF-1 produced by osteoblasts and stromal cells (Figure 1(b)).

## 7. Therapeutic Strategies to Improve Transplantation Outcomes

Accumulating evidence indicates an important role for MMP-2, MMP-9, and MT1-MMP in HSPC mobilization and homing. In particular, modulation of MT1-MMP could become a potential target for development of therapeutic strategies that could improve transplantation outcomes. First, critical to optimizing clinical mobilizing regimens is an understanding of the molecular mechanisms that regulate HSPC mobilization [73, 90, 91]. Such studies have already led to the development of new mobilizing agents that resulted in a rapid collection of more HSPC for transplantation. For example, recent use of Plerixafor (AMD3100) which reversibly binds CXCR4 and disrupts SDF-1—CXCR4 interactions has demonstrated that a combination of AMD3100 and G-CSF results in greater mobilization efficacy compared to G-CSF alone [92, 93].

The main problem with G-CSF-induced mobilization is the variable kinetics of mobilization as shown by the significant number of patients/donors who either mobilize poorly or fail to mobilize. Recent findings indicate that positive regulation of MT1-MMP increases migration/mobilization of HSPC, and hence the development of mobilizing agents that increase MT1-MMP expression could enhance mobilization efficiency. For example, cytokines such as HGF that upregulate MT1-MMP expression in HSPC [11] could also synergize with G-CSF and increase mobilization efficiencies in patients who mobilize poorly with G-CSF alone. This could be tested in a clinical setting following confirmatory results from murine models. On the other hand, potential inhibitors such as green tea polyphenol EGCG, which inhibits MT1-MMP expression and proMMP-2 activation, and statins, which like methyl-*β*-cyclodextrin disrupt lipid raft formation, could inhibit MT1-MMP incorporation into lipid rafts and thereby negatively affect HSPC mobilization. This could result in lowering the number of HSPC collected, and therefore we suggest that overcoming these inhibitory effects could improve transplantation outcomes.

Secondly, a successful transplantation outcome also depends on the ability of a large number of intravenously injected HSPC to rapidly find their way to the BM. This has led to the design of strategies to increase the number of HSPC available by ex vivo expansion of HSPC before transplantation. Another strategy is to enhance the homing potential of a limited number of HSPC by their ex vivo exposure to agents such as C3a and PMV that prime their chemotactic responses by positively regulating the SDF-1—CXCR4 axis, as we previously showed in murine models [40, 87]. Interestingly, this strategy of ex vivo exposure of CB HSPC to C3a is already being evaluated in clinical trial [94]. Therefore we suggest that other priming agents that enhance the responsiveness of CB HSPC towards SDF-1 and upregulate MMPs expression could be used for ex vivo short-term treatment/priming of CB HSPC and be evaluated in clinical trials.

 Lastly, a better understanding of the molecular mechanisms of HSPC migration is not only beneficial for designing novel therapeutic strategies discussed above but could also be applied to enhancement of the homing properties of other types of stem cells such as MSC which share common migration mechanisms.
